# CARE4Kids Study: Endophenotypes of Persistent Post-Concussive Symptoms in Adolescents: Study Rationale and Protocol

**DOI:** 10.1089/neu.2023.0073

**Published:** 2023-12-29

**Authors:** Christopher C. Giza, Gerard Gioia, Lawrence J. Cook, Robert Asarnow, Aliyah Snyder, Talin Babikian, Paul Thompson, Jeffery J. Bazarian, Christopher T. Whitlow, Christopher M. Miles, Scott Otallah, Joshua Kamins, Nyaz Didehbani, Philip E. Rosenbaum, Sara P.D. Chrisman, Christopher G. Vaughan, Munro Cullum, David M. Popoli, Meeryo Choe, Jessica Gill, Emily L. Dennis, Christine L. Mac Donald, Frederick P. Rivara

**Affiliations:** ^1^ Department of Neurology, UCLA School of Medicine, University of California Los Angeles, Los Angeles, California, USA.; ^2^ Department of Neurosurgery, UCLA School of Medicine, University of California Los Angeles, Los Angeles, California, USA.; ^3^ UCLA BrainSPORT Program, Los Angeles, California, USA.; ^4^ Department of Neuropsychology, Children's National Hospital and George Washington University School of Medicine, Washington, DC, USA.; ^5^ Department of Pediatric Critical Care, University of Utah, Salt Lake City, Utah, USA.; ^6^ Department of Psychiatry and Biobehavioral Sciences, UCLA School of Medicine, University of California Los Angeles, Los Angeles, California, USA.; ^7^ Department of Psychology, UCLA School of Medicine, University of California Los Angeles, Los Angeles, California, USA.; ^8^ Departent of Clinical and Health Psychology, University of Florida, Gainesville, Florida, USA.; ^9^ Fixel Institute, University of Florida, Gainesville, Florida, USA.; ^10^ Departments of Ophthalmology, Neurology, Psychiatry and the Behavioral Sciences, and Radiology and Engineering, University of Southern California, Los Angeles, California, USA.; ^11^ Department of Emergency Medicine, University of Rochester School of Medicine and Dentistry, Rochester, New York, USA.; ^12^ Department of Radiology, Wake Forest University School of Medicine, Atrium Health Wake Forest Baptist, Winston-Salem, North Carolina, USA.; ^13^ Department of Biostatistics and Data Science, Wake Forest University School of Medicine, Atrium Health Wake Forest Baptist, Winston-Salem, North Carolina, USA.; ^14^ Department of Family and Community Medicine, Wake Forest University School of Medicine, Atrium Health Wake Forest Baptist, Winston-Salem, North Carolina, USA.; ^15^ Department of Neurology, Wake Forest University School of Medicine, Atrium Health Wake Forest Baptist, Winston-Salem, North Carolina, USA.; ^16^ Department of Psychiatry, University of Texas Southwestern Medical Center, Dallas, Texas, USA.; ^17^ Department of Physical Medicine and Rehabilitation, University of Texas Southwestern Medical Center, Dallas, Texas, USA.; ^18^ Department of Pediatrics, University of Washington School of Medicine University of Washington, Seattle, Washington, USA.; ^19^ Center for Child Health, Behavior and Development, Seattle Children's Research Institute, Seattle, Washington, USA.; ^20^ Children's National Hospital, Washington, DC, USA.; ^21^ Department of Neurology, University of Texas Southwestern Medical Center, Dallas, Texas, USA.; ^22^ Department of Neurosurgery, University of Texas Southwestern Medical Center, Dallas, Texas, USA.; ^23^ Department of Orthopedics and Rehabilitation, Wake Forest University School of Medicine, Atrium Health Wake Forest Baptist, Winston-Salem, North Carolina, USA.; ^24^ Department of Pediatric Neurology, UCLA School of Medicine, University of California Los Angeles, Los Angeles, California, USA.; ^25^ School of Nursing, Johns Hopkins University, Baltimore, Maryland, USA.; ^26^ TBI and Concussion Center, University of Utah, Salt Lake City, Utah, USA.; ^27^ Department of Neurology, University of Utah, Salt Lake City, Utah, USA.; ^28^ Department of Neurological Surgery, University of Washington School of Medicine University of Washington, Seattle, Washington, USA.; ^29^ Department of Epidemiology, University of Washington School of Medicine University of Washington, Seattle, Washington, USA.; ^30^ Center for Child Health, Behavior and Development, Seattle Children's Research Institute, Seattle, Washington, USA.

**Keywords:** adolescent, autonomic nervous system, biomarker, blood, endophenotype, MRI, persistent post-concussion symptoms

## Abstract

Treatment of youth concussion during the acute phase continues to evolve, and this has led to the emergence of guidelines to direct care. While symptoms after concussion typically resolve in 14-28 days, a portion (∼20%) of adolescents endorse persistent post-concussive symptoms (PPCS) beyond normal resolution. This report outlines a study implemented in response to the National Institute of Neurological Diseases and Stroke call for the development and initial clinical validation of objective biological measures to predict risk of PPCS in adolescents. We describe our plans for recruitment of a Development cohort of 11- to 17-year-old youth with concussion, and collection of autonomic, neurocognitive, biofluid, and imaging biomarkers. The most promising of these measures will then be validated in a separate Validation cohort of youth with concussion, and a final, clinically useful algorithm will be developed and disseminated. Upon completion of this study, we will have generated a battery of measures predictive of high risk for PPCS, which will allow for identification and testing of interventions to prevent PPCS in the most high-risk youth.

## Introduction

Concussion is a significant public health issue for children and adolescents—each year in the United States, an estimated 1.1 to 1.9 million individuals under the age of 18 sustain concussions from sports and recreation.^1^ Treatment of youth concussion during the acute phase continues to evolve, and this has led to the emergence of guidelines to direct care.^2–5^ While symptoms after concussion typically resolve in 14-28 days, a portion (∼20%) of adolescents endorse persistent post-concussive symptoms (PPCS) beyond a normal resolution timeline. PPCS is associated with significant disability and diminished quality of life for patients and their families.^6–9^ Efforts to identify objective factors that predict individuals most likely to develop persisting symptoms have been complicated by the use of different definitions of PPCS and diagnostic criteria. Better understanding of these markers is essential to developing targeted, evidence-based treatments to address PPCS.

To this end, the National Institute of Neurological Diseases and Stroke (NINDS) issued a request for applications (RFA-NS020-016) for the “development and initial clinical validation of objective biological measures to be used for prognosing and monitoring recovery of adolescents who either clinically present with or are at risk for developing prolonged/persistent concussive symptoms following exposure to repetitive head impacts and/or concussion. Resultant biological measures should be incorporated into risk stratification algorithms to inform clinical care and patient stratification for future clinical trials.”

This article describes the plan for the study, which is now underway.

### Concept of endophenotypes

Endophenotypes are defined as quantitative biological traits or intermediate phenotypes that can be identified by objective biomarkers using biochemical, radiologic, electrophysiological, molecular, or other techniques.^10^ Such endophenotypes have been used in the psychiatric field to provide a means for decreasing the heterogeneity of patient populations, with a goal of increasing the likelihood of linkages between genetic susceptibility and phenotype. More recently, the term endophenotype or intermediate phenotype has been used in clinical neurology to describe a combination of objective features underlying specific conditions with heterogeneous makeup, such as post-traumatic epilepsy,^10^ chronic traumatic brain injury (TBI),^11^ and dementia.^12^

Prior research in PPCS has used a dichotomous outcome, defining PPCS as the persistence of a minimum of three symptoms for 1 month^13^ or 3 months,^3^ depending on the study, and using a standard tool such as the Post-Concussion Symptom Inventory. However, it is likely that the persistence of post-concussive symptoms such as headache, dizziness, and anxiety have their own unique underlying pathophysiologies, even though they arise from the same injury mechanism. For the purposes of the CARE4Kids Project, an endophenotype is defined as a pattern of quantifiable biomarkers, potentially in combination with objective physical or neurological examination findings and neuropsychological testing results that are linked to the development of PPCS or specific PPCS profiles. A better understanding of the nature of these PPCS endophenotypes is expected to assist the development of more accurate predictors for PPCS. Characterization of these endophenotypes will, thereby, lead to more accurate prognostication and set the stage for future treatment or prevention trials to alleviate the suffering and disability experienced by youth with PPCS.

### Goals and specific aims

The overarching goal of the CARE4Kids Project is to develop a predictive algorithm for PPCS endophenotypes in early and middle adolescents (EMA) to inform clinical screening, management, and future research. This project builds upon existing collaborations to establish a broad, multi-center prospective consortium to prospectively investigate EMA with concussions, with a particular focus on those at risk of developing PPCS. To accomplish these goals, a two-cohort longitudinal study was developed.

#### Aim 1

In a Development Cohort (DC), develop and characterize individual objective biomarkers predictive of PPCS by combining biomarkers with symptom clusters and neuropsychological function. Biomarkers will be examined in three Research Cores: Autonomic Biomarker, Imaging Biomarker, and Blood Biomarker.

#### Aim 2

Using data from the DC, we will develop and characterize endophenotypes of PPCS in EMA by combining two or more objective biomarkers with symptom clusters and neuropsychological measures.

#### Aim 3

We will then collect data from a new sample of EMA with concussion (Validation Cohort or VC) to prospectively validate endophenotype biomarkers from Aims 1 and 2.

#### Aim 4

Using data from the VC, we will create a clinically useful risk stratification algorithm based on validated biomarkers, in conjunction with symptoms and neurobehavioral function, which will predict the development of PPCS.

## Methods

### Overall study design

The CARE4Kids research program uses a two-cohort longitudinal methodology. The DC will undergo testing using the Developmental Test Battery (DTB) of multi-modal biomarkers developed in the three Research Cores. The DC sample will be recruited from multiple sources including emergency departments, urgent care centers, primary care clinics, and specialty clinics. These DC markers were chosen based on differential time course, potential for treatable targets, and longitudinal correlation with PPCS outcome. These biomarkers include autonomic nervous system measurements, MRI neuroimaging, and biofluid tests. The most predictive and scalable DC measures will be incorporated into the Validation Test Battery (VTB) and collected in the second phase, the Validation Cohort (VC). The VC will be recruited from the same Care4Kids consortium sites but expanded to include additional primary care clinics/networks and emergency departments (acutely/subacutely at T1) and followed to 3 months post-initial assessment. In this more generalizable VC, the endophenotype clinical and biomarker measures will be validated as prognostic tools in a risk stratification algorithm, which will set the stage for future studies of treatment for PPCS.

We will also enroll a cohort of 100 typically developing, non-concussed youth ages 11.00-17.99 years who will be used to develop normative data of autonomic function for comparing typically developing youth and youth with concussion.

### Study population, eligibility criteria, and recruitment

The study subjects are enrolled at each of the six sites across the Care4Kids consortium (University of California at Los Angeles, University of Washington/Seattle Children's, Children's National Research Institute, University of Texas Southwestern Medical Center, University of Rochester, and Atrium Health Wake Forest Baptist Hospital). They are being recruited from concussion subspecialty clinics, primary care clinics, emergency departments, athletic trainers who work for the institutions of the investigators, and athletic teams through presentations to teams and parents. Study participants are adolescents of any gender with the following inclusion criteria:

1. Ages 11 to 17.99 years, and
2. Diagnosed with a concussion by a health care provider using Concussion in Sport Group criteria,^5^ and
3. English speaking (parents can be English or Spanish speakers), and
4. Able to be scheduled for the first research visit (T1) < 35 days post-injury, and
5. Continuing to experience post-concussion symptoms at T1, defined as experiencing at least one symptom “more than usual” relative to pre-injury status.

Subjects meeting the inclusion criteria are further screened for any of the following exclusion criteria:

1. Diagnosis of severe autism or other significant developmental delay limiting active participation in the protocol, or
2. Significant neurological disorder (any neurodegenerative disorder, stroke, intracranial hemorrhage, epilepsy) but migraine not excluded, or
3. Severe psychiatric illness or substance abuse (actively suicidal, psychosis), or
4. History of moderate or severe TBI (other than concussion), or
5. Concussion within the last 3 months if asymptomatic at time of evaluation, or any prior concussion if still symptomatic, or
6. Inability to read or sign assent/consent.

In addition to screening at the sites mentioned above, study information is also distributed at local athletic leagues, schools, and community outreach events to raise awareness of the study. After screening subjects, informed consent and assent is obtained either in person or through electronic consent. Approximately 370 subjects will be enrolled during the DC and approximately 350 subjects will be enrolled during the VC; this is further explained below in the section on “Expected power.”

**FIG. 1.**
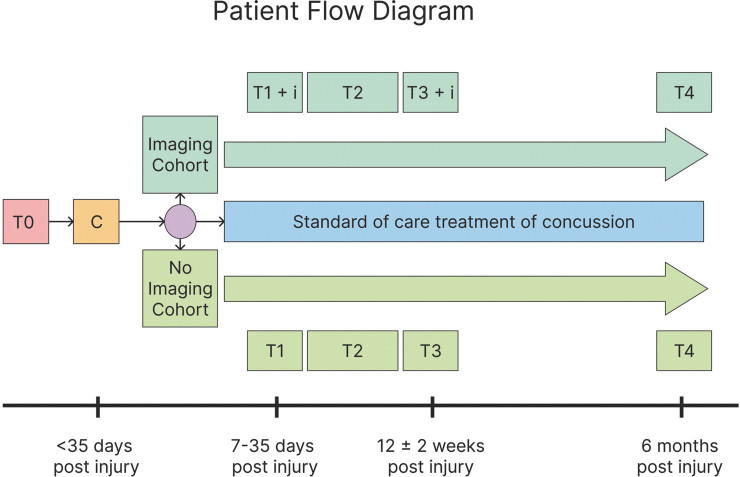
Patient flow diagram for the Discovery Cohort. T0, Date of injury; C, Screen, consent; R, imaging opt out/in; i, neuroimaging conducted; T1, Study visit, labs drawn; T2, Remote engagement with app; T3, Study visit, labs drawn; T4, Remote engagement with app.

Screened and eligible patients are enrolled by research staff using institutional review board–approved parent consent and teen assent forms. Enrolled participants then proceed with the longitudinal research design as outlined in the Participant Flow Diagram (Fig. 1); each of the T1 and T3 assessments takes approximately 5 h to complete. Individuals with metal braces are eligible but will automatically be assigned to the non-imaging group. Financial incentives are offered to both the child and the parent for each visit at T1 and T3; youth completing 6 of 7 days on MyCap (an application that allows outcome data capture via mobile device) are entered into a weekly lottery for an additional $50 incentive.

We will also recruit a control group of typically developing youth ages 11.00-17.99 years who are English speaking but who do not have a history of concussion. The same exclusion criteria for the concussed youth will also apply to them. These individuals will only be studied once and receive the same baseline assessments as the concussed group, but will not receive any MRI examination or blood tests.

### Baseline assessments

**FIG. 2.**
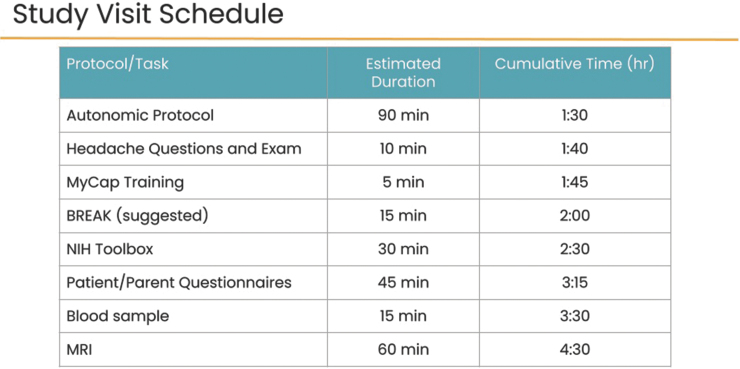
Study visit schedule for the Discovery Cohort.

**FIG. 3.**
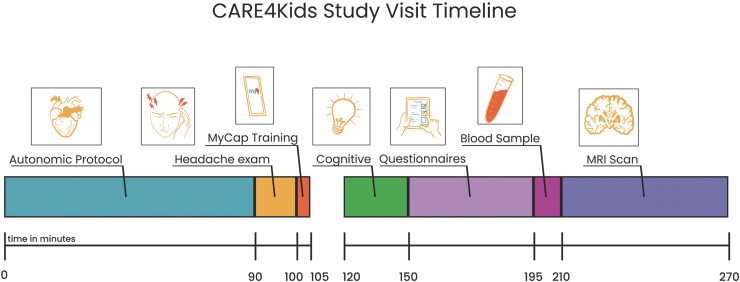
CARE4Kids study visit timeline. Target timeline for a CARE4Kids study visit including a 15-min break.

The baseline assessment (T1) is administered between 7 and 35 days post-injury. Participants undergo the complete test battery, including participant and parent questionnaires, autonomic protocol, headache phenotyping examination, neurocognitive assessment, and blood sample. We also document through parent questionnaire whether other injuries occurred at the time of the event resulting in concussion. Participants undergo MRI neuroimaging at the baseline time-point in addition to the other tests above. A summary of the visit schedule is shown in Figure 2 and the flow through the procedures of the visit is shown as Figure 3.

### Neuropsychological and headache protocol

Persistent cognitive problems following mild TBI (mTBI) in adolescents likely reflect central nervous system (CNS) and autonomic nervous system (ANS) dysfunction, and may be influenced by psychosocial factors, and individual variations in pre-injury function. We therefore collect a rich set of information on premorbid psychosocial function, injury characteristics, and CNS and ANS markers to identify risk factors for adverse cognitive outcomes that can be used to guide timely and effective interventions. The neuropsychological protocol for the study encompasses standardized neurocognitive testing, standardized questionnaires, and a demographic/clinical history questionnaire devised for this protocol.

#### Cognitive measures

The National Institutes of Health (NIH) Toolbox Cognition measures assess the participant's neurocognitive functioning (Table 1A). Fluid, crystallized and overall cognitive abilities will be assessed, including attention/executive functioning, working memory, episodic memory, language, and processing speed. The specific subtests administered include the age-appropriate versions of the Picture Vocabulary Test, List Sorting Working Memory Test, Pattern Comparison Processing Speed Test, Flanker Inhibitory Control and Attention Test, and Auditory Verbal Learning Test. The total time allocated for the Toolbox is approximately 20 min. The NIH Toolbox cognitive measures have shown robust reliability and validity compared to gold standard cognitive measures in children.^14^

**Table 1A.** NIH Toolbox Subtests Administered

Test Name	Type	Domain	Time (min)	Description	Notes
Picture Vocabulary	Crystallized	Language	4	Identify one of four photos that matches the word heard presented	iPad
Flanker Task	Fluid	Attentional Control/Executive	3	Identify the orientation of a fish or an arrow in the middle of the screen and while inhibiting (ignoring) the orientation of the stimuli around it	iPad Home Base
List Sorting	Fluid	Working Memory	7	Recall a list of animals, foods, or foods and animals from smallest to largest	Keyboard and Answer Sheet for RA input
Pattern Comparison	Fluid	Processing Speed	3	Compare 2 pictures and decide if they are the same or different as quickly as possible	iPad
Rey Auditory Verbal Learning	Fluid	Verbal Memory	3	Recall a list of words presented over three trials	RA records responses on iPad

NIH, National Institutes of Health; RA, research assistant.

In coordination with the NIH Toolbox, the Children's Exertional Effects Rating Scale (ChEERS), a 7-item dimensional rating scale of key post-concussion symptoms, will be administered immediately before and after the cognitive tests (NIH Toolbox) to measure changes in symptoms attributable to cognitive exertion.^15^ Exertional effects have been shown to be predictive of outcome above and beyond cognitive performance and Post-Concussion Symptom Inventory (PCSI) symptom ratings.^16^

Symptoms Reports (Table 1B). The primary post-concussion tracking tool in the study is the PCSI,^17,18^ which was designed for patients in our age group. Both self and parent reports are collected at each study time-point (in person and remote visits). At T1, both participant and parents are asked to rate the participant's pre-injury symptoms to allow us to obtain a baseline of symptom load unrelated to injury. In addition, the Post-Concussion Executive Inventory (PCEI), validated for concussion, is administered to both participants and parents to identify real-world deficits in executive functioning. The Pain Catastrophizing Scale (PCS)^19^ modified for concussive symptoms is administered to measure how catastrophizing about concussive symptoms might negatively impact experiences of those symptoms and potentially impact recovery. This measure has been used to identify negative cognitions that predict poor outcome following concussions.^20^ We will also assess the child's exposure to adverse childhood events (ACEs), using the adolescent self-report Pediatric ACEs and Related Life-events Screener (PEARLS). Additional measures of personal, psychological, and family factors to measure the biopsychosocial tapestry of concussion risk are administered (see Tables 1A and 1B for full list of measures). The participant questionnaires take approximately 35-45 min to administer. The parent questionnaires take approximately 30 min to complete.

**Table 1B.** Neuropsychological Battery Administered

Measure	Self (min)	Parent (min)	# items	Type
Post-Concussion Symptom Inventory (PCSI)	4	4		Set
Post-Concussion Executive Inventory (PCEI)	4	4		Set
Short STAI (State-Trait Anxiety Inventory), State	1	–	6	Set
K-CAT (psychiatric symptoms)	7	5		CAT
Pain Catastrophizing Scale (modified)	2	2	13	Set
CLASS-3 (school probs and stress response)	4	4		Set
PROMIS - Sleep	2	2	4 or 10	CAT
Demographic/Clinical History	4	5	variable	Set
Pubertal Development Scale (PDS)	2	–		Set
HIT 6 (migraine severity and PEDS MIDAS (migraine related quality of life)	2	–		Set
Multi-dimensional Assessment of Parenting Style (MAPS)	–	5	34	Set
Parenting Style Inventory-II (PSI-II)	4	–	15	Set
Connor-Davidson Resilience Scale	4	–	25	Set
PACE Self-Efficacy	4	–	17	Set
Family Resources Scale (FRS)	–	5		Set
Adverse Childhood Experiences (ACEs)		2		Set
Minutes (total)	44	38		

MAPS measures family functioning (e.g., dimensions of warmth, supportiveness, hostility, etc.).

CAT, computer adapted test.

### Headache

This portion of the study is targeted towards better defining endophenotypes leading to post-traumatic headache (PTH). Classification based on current International Classification of Headache Disorders-3 criteria (which only broadly classifies post-traumatic headache)^21^ may not reflect complexities of PTH pathophysiology in the setting of concussion. A better understanding of PTH and associated neurological dysfunction may require a finer granularity of phenotypic characterization—distinct from what has been developed for primary headache disorders. Determination of PTH phenotype may have practical importance not only in terms of predicting persisting symptoms, but also as an indicator of potential benefit of phenotype-specific therapies.

We postulate that migraine-like post-traumatic headache may be a unique post-concussion endophenotype with a distinct recovery timeline and markers that could be inferred from physical, imaging, autonomic or blood biomarkers. There are numerous components that will test this hypothesis. Extensive headache questionnaires are obtained at the T1 and T3 visits (Table 2). Headache questions were designed to integrate with the Headache and Concussion NINDS Common Data Elements (CDEs). We also chose to use the MyCap platform^22^ to longitudinally monitor headache and associated features, medication use, and a basic indicator of sleep quantity to more fully characterize headache burden and characteristics from recruitment and the first study visit (T1) until the recovery visit (T3). We are also utilizing validated migraine disability assessment tools at the T1 and T3 research visits, the Pediatric Migraine Disability Assessment (PedsMIDAS),^23,24^ and Headache Impact Test 6 (HIT 6).^25^

**Table 2.** Symptoms and Examination Checklist for Headache Assessment

T1 symptoms:	
Presence of headache	Yes/no
Headache temporal features	Onset of headache after injury (days), frequency (or continuous)
Headache Severity	1-10 pain scale
Headache Quality	Pulsating/constant/stabbing
Headache Location	Unilateral/bilateral, frontal, temporal, parietal, occipital, vertex, global
Associated symptoms	Light/sound sensitivity, Nausea/anorexia
Migraine aura symptoms	Vision, sensory, language, motor symptoms
Cranial autonomic symptoms	Eye redness/tearing, nasal discharge
Other associated symptoms	Cognitive dysfunction, fatigue
Neck pain	With or without headache
Dizziness, vertigo, and/or lightheadedness	With or without headache
Vision	Blurred vision, double vision, difficulty focusing, difficulty reading
Exacerbating/relieving	Movement/activity, upright vs. supine posture, menstrual period if applicable
Prior headache history	Previous history of migraine diagnosis/ severe headache of any kind
Family history	Family history of migraine diagnosis/severe headache of any kind
Medication use	Analgesics, oral contraceptives, stimulants, antidepressants
Caffeine use	
Cannabis use	
Examination	
Vital signs	
Screening neurological exam	Evaluation of eye movements
Test of convergence function	Normal/abnormal
Occipital nerve tenderness	Yes/no
Anterior radiation of pain with occipital nerve pressure	Yes/no
Ipsilateral/contralateral neck pain on flexion, extension, rotation w/extension (facet load)	Yes/no

Headache Impact/Disability Metric: Headache Impact Test – 6 (HIT-6)	
Between T1 and T3 - Daily Phone Diary:	
Presence/absence of headache	Yes/no
Duration of headache	Hours or continuous
Headache severity	1-10 scale
Location of headache	
Nausea	Yes/no
Light or sound sensitivity	Yes/no
Dizziness or lightheadedness	Yes/no
Blurred or double vision	Yes/no
Flashing lights or wavy lines in vision	Yes/no
Medication intake	Specify
Sleep	Hours

T3 (second in-person visit)	
Clinical features: Same as T1 without historical features	
Examination: same as T1	

Associated with these headache features, we are characterizing blood-flow pathophysiology through advanced neuroimaging (arterial spin labeling; ASL) and white matter microstructure with diffusion tensor imaging and quantitative susceptibility mapping (QSM). The prospective measurement of cerebral blood flow (CBF) using ASL in this project has the potential to provide highly meaningful information regarding changes in blood flow as a mechanism of PTH and as an indicator of PPCS outcomes. Fluid biomarker data will also supplement this analysis, we are investigating pathophysiologic associations with pain neuropeptides such as calcitonin gene-related peptide, pituitary adenylate cyclase-activating polypeptide, and Substance P.

To assess for physical examination markers that correlate with outcomes, research coordinators have been trained in a standardized head and neck examination, tailored for headache disorders, to assess for cervical and neurologic components of persistent posttraumatic headache (Table 2).

### Autonomic biomarker protocol

There is growing interest in the role of the autonomic nervous system (ANS) in concussion because of major overlap in symptoms of concussion and dysautonomia. Many patients with concussion present with classic dysautonomic signs such as postural orthostatic tachycardia, headache, nausea, dizziness, fatigue, and anxiety.^26–29^ Conversely, many patients with autonomic disorders without brain injuries have similar symptoms as patients with concussion, including dizziness, vertigo, nausea, mood swings, anxiety, fatigue and intolerance to exercise, concentration and memory problems, and photophobia and phonophobia.^5^ Brain regions that may be particularly vulnerable to head impact forces, such as limbic cortex, hypothalamus, and midbrain nuclei, are all key structures in the central autonomic network. In a recent review, 33 of 36 studies identified ANS anomalies in concussed athletes not found in non-athletes.^26^ Given the dearth of treatments for persistent concussion symptoms, autonomic dysregulation may be a promising target for new mechanistically grounded treatments.

Given the limitations in prior research, we designed a protocol to measure psychophysiological responses (heart rate, blood pressure and respiratory dynamics) under conditions shown in prior research to activate sympathetic and/or parasympathetic activity. In addition, we include a non-cardiovascular measure of autonomic function-the pupillary light response. A potentially important innovation is the use of an inexpensive wearable device (Scosche Armband)^30^ to collect heart rate data in parallel with one of the most widely used electrocardiography systems to assess cardiac function in autonomic function research—the Biopac system. If the data from the Schosche Armband provide heart rate data comparable to the Biopac system we will consider using it in the validation phase.

**FIG. 4.**
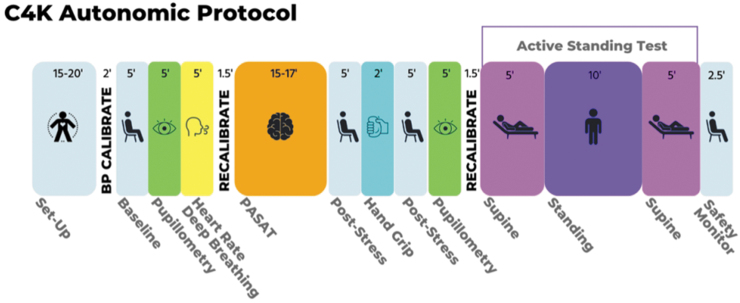
Autonomic protocol.

The autonomic assessment protocol continuously measures psychophysiological responses during several conditions designed to stimulate sympathetic and/or parasympathetic activity. The conditions (Fig. 4) include heart-rate deep breathing, pupillary light response, cognitive/emotional challenge (Paced Auditory Serial Addition Test), hand-grip test, and an active standing test. Each of the stressors is followed by a post-activation recovery period to measure the stress-response and recovery. The protocol is complicated, requiring presentation of instructions to participants about how to respond to a variety of tasks and different stimuli. To standardize the administration of instructions, presentation of stimuli, and acquisition of psychophysiological data across six data collection sites, stimulus presentation software (E-Prime) is used to deliver the instructions to participants, as well as the visual and auditory stimuli for all conditions, task timing and recording response. This approach reduces the burden on research staff allowing them to focus on ensuring proper signal acquisition. E-Prime directly interfaces with continuous data acquisition software (Biopac AcqKnowledge) and automatically time-locks task events. The Scosche Band collects data concurrently, but on a separate application.

Human factors were strongly considered given the challenge of training research staff at six sites, anticipated staff turnover, and the need for ongoing quality control. To support research staff in navigating the detailed training and competency required for this project, all training information is housed on an interactive, online smart board, Miro.^31^ This website allows centralized hosting of training manuals, videos demonstrations, informational documents, updates to the protocol, and monitoring systems easily accessible through a visual format, which we believe facilitates training and protocol fidelity across sites. Research assistants undergo extensive training in how to connect electrodes, blood pressure cuffs, etc., to participants to acquire biologically accurate signals, and protocol administration using E-Prime. Since research sites are distributed across the country, experiment fidelity and data quality are ensured by verifying signal accuracy of the psychophysiological data and then by multiple live observation of study protocols conducted via zoom. This allows the research coordinators at UCLA to monitor both the participant and the data coming in.

### Imaging biomarker protocol

Multi-modal MRI can reveal and measure a wide spectrum of brain alterations in mTBI, beyond gross anatomical changes (Table 3). Diffusion MRI (dMRI) can model white matter (WM) tracts, and is acutely sensitive to microstructural alterations that result from mTBI, including axonal damage, glial and inflammatory responses, and edema.^32^ Some studies using dMRI after sports related concussion show poorer WM organization after injury, correlated with plasma biomarkers of injury-related inflammatory processes.

**Table 3.** Imaging Modalities

Modality	Scan parameters	Derived Measures	Usefulness
Anatomical MRI (T1w and T2w)	Voxel size = 1 mm^3^, 176-225 slices (T1w), 176-256 slices (T2w), scan time = 9-14 min for both	Regional cortical thickness and surface area, subcortical volumes for 96 regions	Morphometry can be altered in children with concussion and TBI relative to neurotypical controls
dMRI	Voxel size = 1.7mm^3^, diffusion weighting = 500 (6), 1000 (15), 2000 (15), 3000 (60), 81 slices, scan time = 7-9 min	Standard (tensor) and advanced DKI and NODDI metrics of WM microstructure; diffusivity metrics for 14 major WM tracts	Examines tracts that have previously shown atypicality in children with concussion and TBI
SWI/QSM	Voxel size = 0.5-1.0 × 0.5-1.0 × 2.0-3.0 mm^3^, scan time = 6 min	Magnetic susceptibility of biological tissue; regional T2^*^ values	Reflects venous vasculature, hemosiderin microbleeds, and aspects of microstructure (e.g., iron, calcium, and myelin).
rsfMRI	Voxel size = 2.4 mm^3^, 60 slices, TR = 800 msec, TE = 30 msec, scan time = 20 min (4 × 5 min runs)	Functional connectivity metrics using seeds from the 20-component analysis of the BrainMap activation database and rsfMRI dataset	Examines functional activity and connectivity
ASL	Voxel size = 1.8 × 1.8 × 4.0 mm^3^, 26 slices, scan time = 5 min	Regional measures of cerebral blood flow	Measures perfusion

MRI, magnetic resonance imaging; TBI, traumatic brain injury; dMRI, diffusion MRI; DKI, diffusion kurtosis imaging; NODDI, Neurite Orientation Dispersion and Density Imaging; WM, white matter; SWI, susceptibility-weighted imaging; QSM, quantitative susceptibility mapping; rsfMRI, resting-state functional MRI; TR, repetition time; TE, echo time; ASL, arterial spin labeling.

The timing of assessment is critical for dMRI, however, as swelling and bleeding can alter measures.^33^ With resting-state functional MRI (rsfMRI), researchers can quantify disruptions in coordinated functional activity across the brain. In acute mTBI, alterations in functional connectivity have been detected,^34,35^ and may be predictive of cognitive performance months later.^34^ Similar results have been shown in sports-related mTBI, with acute alterations that resolved later.^36,37^ Although rsfMRI studies can identify disruptions in acute mTBI, challenges in interpreting the blood oxygen–level dependent signal mean that rsfMRI on its own cannot determine whether disruptions are due to disconnection (site or network specific), poor perfusion, disruption of cerebrovascular reactivity, or a hybrid of these.^38^ ASL maps changes in CBF at a fine scale across the brain, revealing direct vascular contributions of rsfMRI alterations. To date however, studies of ASL in mTBI have been inconclusive: some studies show lower CBF 24-48 h after injury^39^ while others show higher CBF (2 and 6 weeks post-injury),^40^ potentially with an interaction with sex.^41^ Lastly, QSM is modality whose application to TBI has accelerated in the last decade, aiming to noninvasively estimate the magnetic susceptibility of biological tissue.^42^ QSM noninvasively estimates the magnetic susceptibility of biological tissue, which can be altered by macro- or micro-hemorrhage. QSM images can also be used to derive quantitative measures of white matter hyperintensity burden and for qualitative grading of lacunar infarctions and evidence of closed head injury (e.g., cortical contusions). In the CARE4Kids project, we modeled our protocols on the Adolescent Brain Cognitive Development (ABCD) neuroimaging protocol which has been successfully used to scan over 10,000 adolescents,^43^ supplemented with specialized scans ideal for clinical assessment of concussion (ASL and QSM).

### Fluid biomarker protocol

Several key physiologic processes likely related to the persistence of PPCS are detectable in peripheral blood. Concussion-related axonal injury, inflammatory activity, membrane turnover and oxidative stress result in altered neurotransmission leading to a disruption of neural networks.^44^ These physiologic processes can be assessed in peripheral blood by measuring neuronal/glial proteins, lipids, inflammatory cytokines, and neuropeptide neurotransmitters. A group of blood-based proteins reflecting these processes was selected based on evidence of diagnostic and/or prognostic capability and availability of commercial assays (Table 4). While most published blood biomarker studies involve adults, several involving EMAs underscore their potential in this age group.^45-47^

**Table 4.** Proposed Fluid-Based Biomarkers to be Measured in EMAs

TBI Physiologic process	Marker Type	Marker
Axonal injury	Neuronal/glial Injury	GFAP, UCH-L1, total Tau, NF-L
Inflammatory activity	Cytokines	TNF-α, IL-6, IL-8, IL-10, VEGF
Membrane turnover and oxidative stress	Lipids	LPA, FA 2-OH C16:0, FA C18:0, TUDCA, PE ae C36:4, PE aa C38:6, LysoPC a C20:4
Altered neurotransmission	Neuropeptides	CGRP, PACAP, Substance P

EMA, early and middle adolescents; GFAP, glial fibrillary acidic protein; UCH-L1, ubiquitin carboxy-terminal hydrolase isoenzyme L1; NF-L, neurofilament light chain; TNF-α, tumor necrosis factor alpha; IL, interleukin; VEGF, vascular endothelial growth factor; LPA, lysophosphatidic acid; FA 2-OH C16:0, 2-hydroxypalmitic acid; FA C18:0, stearic acid; TUDCA, tauroursodeoxycholic acid; PE ae C36:4, phosphatidylethanolamine plasmalogen; PE aa C38:6, diacyl-phosphatidylethanolamine; LysoPC a C20:4, lysophosphatidylcholine; CGRP, calcitonin gene-related peptide; PACAP, pituitary adenylate cyclase-activating polypeptide.

Quantification of fluid biomarkers (FBMs) at a single, acute post-injury time-point, as well as changes across two time-points, can aid in the early identification of EMAs most vulnerable to PPCS. Importantly, these biomarkers have different temporal dynamics,^48^ which suggests that a combination of these markers (or changes in marker values over time) might be the most effective in identifying EMAs at risk for poor recovery and chronic symptoms.

Using markers in blood to assess physiologic vulnerability to PPCS in EMAs has several advantages. The processes of drawing, shipping, and storing blood are well known world-wide and the equipment required relatively inexpensive. In addition, blood-based protein assays are readily scalable. The blood drawing procedure is well known and accepted by the general public, although EMAs may be more likely to be adversely affected by the anticipatory anxiety and distress associated with venipuncture.^49^ To address this barrier, a needle-free, lancet-based device shown to improve the acceptability of phlebotomy in pediatric populations^50,5^1 is also be tested as part of this protocol.

Non-fasting blood samples will be collected at T1 and T3 by certified phlebotomists via venipuncture using specimen collection kits provided by the NINDS BioSEND Repository following established protocols. Serum and plasma will be collected from whole blood following BioSEND standard processing procedures. Within 30 min of collection, samples will be centrifuged at 2000 G for 15 min and stored locally at -80°C and then shipped overnight on dry ice to BioSEND Repository for long-term storage. Assays will be batched to minimize variability, with each batch run with appropriate standards and controls to ensure reliability.

It is anticipated that integration of FBMs, along with imaging and autonomic biomarkers, into a clinically practical risk-stratification algorithm will be critical for accurate prediction of EMAs at high risk for PPCS.

### Remote assessment—T2

Following the T1 test battery, participants and parents will respond to questionnaires from home using their mobile devices. This study employs MyCap, a mobile application developed by Vanderbilt University, that integrates with the REDCap study database. A mobile device will be provided to those parents and/or participants who do not have one of their own. Over the approximately 2-month period between the T1 and T3 test batteries, participants and parents will receive the following questionnaires:

Participants:

1. Daily questionnaires querying headache phenotype and exercise
2. Weekly questionnaires querying additional behaviors such as napping and stress
3. Bi-weekly administration of the PCSI

Parents:

1. Bi-weekly questionnaire about changes in medications, treatment, and other interventions
2. Bi-weekly administration of the Parent-PCSI

T2 questionnaire distribution begins starting the day after T1 and proceeds until the T3 timepoint (3 months post-injury).

### Three-month follow-up assessment—T3

Three months after injury, the participants complete the entire test battery administered during the baseline assessment, T1. Participants in the imaging arm will undergo neuroimaging again at this time-point. It is anticipated that some participants will not be willing or able to return for an in-person assessment at this time. For those participants, a remote T3 is available, which includes questionnaires but without biomarker assessments. All participants are encouraged to attend the T3 in person for full data acquisition.

### Recovery discovery assessment—T4

To collect complete recovery data, parents will receive monthly questionnaires at 4-, 5-, and 6-months post-injury to ask whether their child has recovered from the concussion. Once the parent indicates complete recovery, which is defined for them as “all of the symptoms that were caused BY THE INJURY have GONE AWAY and DO NOT RETURN when doing physical or mental activities such as exercise or studying for school,” they will estimate a date of recovery and will no longer receive surveys. Parents who indicate “no, not recovered” will complete additional questions about the nature of the persistent symptoms and will continue to receive monthly queries up to 6-months post-injury.

### Data analysis

#### Development cohort analysis

##### Analytical approach

Our overall approach will involve iteratively fitting models to predict PPCS as defined by a PCSI Retrospective Adjusted Post-Injury Difference (RAPID) score (pre-injury PCSI score subtracted from the post-injury PCSI score) greater than 5 at the T3 visit.^52^ Benchmark predictability will be determined with clinical factors alone; next we will test predictability of individual biomarkers alone; the third step will combine clinical, biomarker and psychosocial variables; finally, we will examine the effects of confounders and effect moderators such as medication and treatment, gender, mechanism of injury, and comorbidities. For each model we will estimate sensitivity, specificity, positive predictive value (PPV), negative predictive value (NPV), and area under the receiver operating characteristics curve (AUC). We will employ 10-fold cross validation to avoid overfitting. Point estimates will be the average of the estimates from each of the ten folds and we will calculate bootstrapped confidence intervals.

##### Benchmark model

Clinical variables previously shown to be related to developing PPCS^13^ include: age, gender, prior concussion history and symptom duration, history of physician-diagnosed migraine, physician-diagnosed anxiety, physician-diagnosed depression, self-reported headache, self-reported sensitivity to noise, and self-reported fatigue. We will test for differences between participants with and without PPCS using chi-squared or Fisher's exact tests as appropriate. We will fit separate logistic regression models using each variable as well as varying combinations of variables. Models will be ranked in terms of AUC with the model producing the best testing characteristics serving as the benchmark for evaluating improvements at predicting PPCS with the addition of biomarkers.

##### Individual biomarkers

For each biomarker, the AUC and Youden's index will be calculated in univariable logistic regression models to predict PPCS. One model per imaging modality will be developed. For modalities producing many predictors, we will use principal components analysis (PCA) to reduce dimensionality prior to modeling. The optimal dichotomization of each multi-level or continuous biomarker will be selected using a combination of Youden's index and clinical judgment.

##### Benchmark + individual biomarkers

Next, we will separately add each biomarker and psychosocial variables to the benchmark model and assess changes in testing characteristics. Biomarkers will be ranked from most to least improvement in AUC. Any biomarker with a marginally significant *p* value (*p* < 0.20) for either a main effect or interaction will be eligible for the risk stratification model and continued in the Validation Phase.

##### Expected power

We will enroll 370 EMA in the Development Cohort. With an expected loss to follow-up rate of 15%, we project having data from 315 EMA available for this analysis. We project 35% of males and 50% of females to have PPCS at follow-up.^53^ Assuming an equal split between males and females, we estimate we will have 268 PPCS participants in the Development Cohort. Based on the results of the 5P study, we expect our benchmark model to achieve an AUC of 0.68. Our expected sample size will provide a power of 0.78 to detect an increase in AUC of 0.06.

##### Risk stratification algorithm

Using all clinical factors and biomarker passing the above threshold, we will develop a risk stratification tool for predicting PPCS. A common rule of thumb for logistic regression models is to have at least 10 participants per predictor. It is likely we will have more than 26 clinical and biomarker candidate variables; therefore, we will apply a least absolute shrinkage and selection operator (LASSO) penalty to our logistic regression model. We will use 10-fold cross validation to estimate the optimal value of the tuning parameter, t*. We will then choose the value of t, t′, which produces the least complex model and has a prediction error within one standard deviation of the t* model. Our clinical risk score will be developed from our final model by assigning points to each predictor variable with the final point total corresponding to the risk estimate, following the method described by Sullivan and colleagues.^54^ High, medium, and low risk cut points will be determined by a consensus meeting of the investigators. We will report standard testing metrics such as AUC, sensitivity, specificity, PPV, NPV, likelihood ratio negative, and likelihood ratio positive.

### Validation cohort analysis

We will use the Validation Cohort to test the predictive ability, sensitivity, and specificity of our algorithm. If we are unable to demonstrate acceptable performance of the risk stratification rule (AUC <0.65) on the validation cohort, we will create a new algorithm which incorporates 66% of the development and validation cohorts for derivation and validate it on the remaining 34% of participants.

### Endophenotypes

Latent class analysis (LCA) will be used to classify multi-dimensional clinical and biomarker variables into phenotypic groups. Continuous variables and biomarkers will be dichotomized or broken into three groups (i.e., high, medium, low) according to clinical and biological cutoffs. To avoid fitting a model with too many variables we will conduct screening on all potential clinical, biospecimen, autonomic, and imaging variables. We will compare distributions of predictors between the PPCS and non-PPCS groups using two-sample t-tests or Wilcoxon rank sum tests for continuous predictors and chi-square tests or Fisher's exact tests for categorical and binary predictors. Predictors achieving a marginally significant *p* value of 0.10 or less will be included in the LCA model. The number of classes will be selected by comparing the log likelihood ratio, Bayesian Information Criterion (BIC), and *p* values. Models producing sparse groups will be rejected in favor of models with fewer groups of sufficient size. Models will be assessed for clinical plausibility and goodness of fit will be examined using entropy r-squared, univariate residuals, and bivariate residuals.

If too many variables pass the screening method and threaten the stability of the LCA model, we will first perform dimension reduction via PCA within each domain: clinical, FBM, autonomic, and imaging. We will investigate the use of previously published scores, as well as perform PCA within domains before rerunning the LCA. In order to capture any interactions potentially excluded from the LCA approach, we will build a Classification and Regression Tree (CART). Different paths through the tree will be examined for combinations of variables and identification of phenotypes. Ten-fold cross validation will be used to determine the optimal value for the complexity parameter. Variables determined to be most important for class selection will be examined. Any biomarkers important for identifying phenotypes will be continued in the Validation Phase. This analysis will be repeated for two specific endophenotypes of interest migrainous headache and anxiety/mood disorders by replacing the general outcome of overall PPCS with each specific symptom cluster.

As an alternate approach, we will apply a group LASSO procedure where categories of predictors are included or excluded together. In this fashion, we will be able to assess the importance of each type of biomarker or clinical variable. Additionally, LASSO can sometimes perform poorly in the presence of many correlated predictors. We will explore utilizing an elastic net procured for deriving our final logistic regression model as well. All models will be compared in terms of AUC and sensitivity.

### Anticipated products

There are several major deliverables that will arise from this project. The primary goal will be an algorithm to improve prediction of one or more endophenotypes of PPCS. By linking clinical symptom phenotypes to objective markers (neurocognitive, PTH, autonomic, imaging and/or molecular), it is hypothesized that different biological categories of PPCS will be identifiable as early as possible. This will have implications for clinical management: 1) mechanism-based therapies may be directed toward specific endophenotypes; and 2) different endophenotypes may have different prognosis and recovery trajectories. Further, the potential to identify these subtypes of PPCS will likely enhance research efforts by focusing future clinical treatment trials to endophenotypes with specific biological targets, as well as open the possibility of preventive interventions to interrupt ongoing processes that may lead to chronic symptoms.

This effort will also contribute substantially to the Federal Interagency Traumatic Brain Injury Research (FITBIR) database, providing not only clinical data (symptoms, neurologic examination and neurocognitive) but linked to deep biomarker phenotypes based on each of the three cores. Importantly, much of the extant data in FITBIR comes from adult TBI subjects (TRACK-TBI, CARE Consortium), and a major contribution of CARE4KIDS CONSORTIUM will be the addition of hundreds of adolescent mTBI/concussion subjects. In addition to the repository of individual multimodal data, CARE4KIDS CONSORTIUM will also collect, organize, and store individual blood/serum samples in the NINDS biorepository at BioSEND. This will be a resource for future investigations.

Another important contribution of CARE4Kids Consortium will be the innovative use of different remote data collection methods. This study is collecting interim symptom and treatment data from both subjects and parents during the remote T2 data collection, using prompts from the MyCap app, which interfaces with the REDCap database. In addition, we are monitoring data entry compliance and using compliance as a retention/motivational tool to keep subjects engaged between their in-person T1 and T3 study visits. We are also using a follow-up questionnaire, Recovery Discovery (piloted during the 4CYC data collection that preceded CARE4Kids Consortium) to help identify more precisely when a subject symptomatically recovers from their concussion. Remote blood collection will be explored through a comparative study of the Hemolink device with conventional venipuncture. If validated, this could provide an important additional means to capture blood-based markers without the subjects having to be in-person. And lastly, while the autonomic biomarker core currently requires a complex, highly monitored data collection protocol, promising remote autonomic data collection devices (like the Scosche armband) will be validated against a gold standard of in-person physiological monitoring. Such devices offer the potential to collect daily changes in activity, sleep, and autonomic function.

## Discussion

As noted above, the clinical implications of identifying key predictive factors that can lead to PPCS become important to identify early post-injury to reduce or even eliminate undue suffering or disability. Ultimately a definition of the endophenotypes predicting prolonged recovery will provide information for the clinician to activate early surveillance of the patient's recovery profile and the initiation of active treatments to ameliorate the issues. This early definition of the patient's clinical needs will facilitate a more confident and systematic approach to recovery management. The next step, however, is to define the targeted evidence-based treatments through clinical trials. For example, the definition of autonomic factors that contribute to the presence of persisting headaches or neuropsychological factors that underlie post-concussive anxiety provide key areas toward which treatments can be directed early in recovery.

The approach to concussion management has been evolving from nonspecific treatments (rest, exercise, psychoeducation, sleep) to a more tailored strategy based upon the patient's predominant symptoms. This older approach may have contributed to the chronicity of “post-concussion syndrome” and offered little in the way of specific interventions. It also created limitations on the ability to design controlled treatment trials. Subtypes of concussion have begun to be delineated, with the goal of more treatment individualization; however, current subtyping still relies predominantly on subjective self-reported symptoms.^55,56^ Adding objective measures to concussion subtypes, and particularly PPCS subtypes, is hypothesized to lead to more selective treatments and improved prognosis. Other neurological diagnoses have begun examining the concept of combining clinical signs and symptoms with more objective markers of underlying biology to develop endophenotypes or endotypes of post-traumatic epilepsy or neurodegeneration.^57^ By obtaining objective neurobiologically-based endophenotypes of those with PPCS, the CARE4Kids study will help elucidate different biological processes that may contribute to persisting symptoms after concussion, and also lay groundwork for more mechanism-based therapeutics for those experiencing long-term suffering and impairments.

## Transparency, Rigor, and Reproducibility Summary

This is a protocol for a pathophysiological mechanistic study. This study was not formally registered because it is not an intervention trial. This manuscript serves as the methods paper for the project to publish the methodology and protocol. The analysis plan is pre-specified as outlined in this methods manuscript. Statistical power and sample size calculations were conducted which informs the enrollment targets. No consort diagram is provided in this article since it is describing the methods of the study rather than any data or analysis. Subjects are blinded to many of the results of the experimental manipulations; the exception of results that will be shared with the subject are shared after the final observations are made for the study visit. The measures utilized in this protocol are validated measures. Data from this project will be shared with the FITBIR repository. The authors agree to provide the full content of the manuscript on request by contacting the corresponding author listed below.
